# Exploring the Antioxidant and Genoprotective Potential of *Salicornia ramosissima* Incorporation in the Diet of the European Seabass (*Dicentrarchus labrax*)

**DOI:** 10.3390/ani14010093

**Published:** 2023-12-27

**Authors:** Raquel Marçal, Pedro Sousa, Ana Marques, Vitória Pereira, Sofia Guilherme, André Barreto, Benjamin Costas, Rui J. M. Rocha, Mário Pacheco

**Affiliations:** 1 CESAM—Centre for Environmental and Marine Studies and Department of Biology, University of Aveiro, 3810-193 Aveiro, Portugal; sousa.p@ua.pt (P.S.); anammarques@ua.pt (A.M.); vitoria.pereira@ua.pt (V.P.); sofia.g.guilherme@ua.pt (S.G.); mpacheco@ua.pt (M.P.); 2 Riasearch, Lda., 3870-168 Murtosa, Portugal; andrebarreto@riasearch.pt (A.B.); ruirocha@riasearch.pt (R.J.M.R.); 3 Centro Interdisciplinar de Investigação Marinha e Ambiental (CIIMAR), Universidade do Porto, 4450-208 Matosinhos, Portugal; bcostas@ciimar.up.pt; 4 School of Medicine and Biomedical Sciences (ICBAS-UP), University of Porto, 4050-313 Porto, Portugal

**Keywords:** halophytes, functional feed, DNA integrity, sustainable aquaculture, fish feedstuff

## Abstract

**Simple Summary:**

*Salicornia ramosissima* was studied as a potential feed material for juvenile European seabass in aquaculture. This halophyte was incorporated in three different levels (2.5, 5 and 10%) into the fish diet for two months. The results showed no negative impact on the fish’s growth, and there was an improvement in antioxidant activity in specific tissues, particularly through the GSH-related defense subsystem. A genotoxic trigger was also identified in the first month, which was considered a protective mechanism, and in the second month, a clear protection of DNA integrity was observed, especially at higher supplementation levels. Overall, the findings suggest that supplemented diets with *S. ramosissima* have functional benefits, offering a potential way to enhance aquaculture practices and utilize a novel, low-value raw material.

**Abstract:**

The identification of novel feed materials as a source of functional ingredients is a topical priority in the finfish aquaculture sector. Due to the agrotechnical practices associated and phytochemical profiling, halophytes emerge as a new source of feedstuff for aquafeeds, with the potential to boost productivity and environmental sustainability. Therefore, the present study aimed to assess the potential of *Salicornia ramosissima* incorporation (2.5, 5, and 10%), for 2 months, in the diet of juvenile European seabass, seeking antioxidant (in the liver, gills, and blood) and genoprotective (DNA and chromosomal integrity in blood) benefits. Halophyte inclusion showed no impairments on growth performance. Moreover, a tissue-specific antioxidant improvement was apparent, namely through the GSH-related defense subsystem, but revealing multiple and complex mechanisms. A genotoxic trigger (regarded as a pro-genoprotective mechanism) was identified in the first month of supplementation. A clear protection of DNA integrity was detected in the second month, for all the supplementation levels (and the most prominent melioration at 10%). Overall, these results pointed out a functionality of *S. ramosissima*-supplemented diets and a promising way to improve aquaculture practices, also unraveling a complementary novel, low-value raw material, and a path to its valorization.

## 1. Introduction

Arable lands are diminishing globally because of soil salinization as well as freshwater scarcity due to the lack of precipitation and improper water resource management practices [1]. Thus, the use of salt-tolerant plants (halophytes) to produce food and feed emerges as a sound and timely alternative to conventional crop production, overcoming constraints such as the limited adoption of sustainable and responsible agricultural practices [2], coupled with competition with direct human consumption. In this direction, the cultivation of halophytes is gaining recognition and, recently, its valorization as a food product and novel feedstock for nutraceuticals [3] and pharmaceutical [4] applications has been investigated (revised by [5]).

These premises also make halophytes good candidates as a new source of feedstuff for aquafeeds, with potential economic viability and gains in terms of environmental sustainability. However, investigations in this direction are scarce and completely absent in carnivorous fish species. To our knowledge, only a couple of studies have been carried out addressing the replacement of fish meal with *Salicornia bigelovii* in Nile tilapia (*Oreochromis niloticus*) feed [6,7], showing no compromise to growth parameters or body composition. Therefore, research and development efforts focused on this topic appear perfectly justifiable, innovative, and opportune, namely concerning high-value aquaculture species, mainly represented in the European context by marine teleosts (e.g., salmon, seabream, and seabass).

The halophyte *Salicornia ramosissima* is an annual green tip plant that can be found in saltmarshes, with a broad geographical distribution (from Artic to Mediterranean regions), characterized by a pleasant texture, a juicy and salty taste [8], as well as phytochemical profiling that supports its edibility and food relevance [9]. Beyond the traditional culinary applications, *Salicornia* spp. are rich in fibers, minerals, polyphenols, flavonoids, and proteins, and poor in fat, emerging thus as valuable nutritional and healthy food for humans [10,11,12,13]. These halophytes also showed medicinal attributes, namely that they are immunomodulatory, lipid- and sugar-lowering, antiproliferative, and osteoprotective, as well as reinforcing antioxidant and DNA repair systems [9,12,13,14]. Bearing in mind these properties, *Salicornia* spp. should be cogitated as a promising supplement ingredient for inclusion in feeds for marine fish farming.

The major challenge of seeking novel feed supplements/materials is the combined achievement of environmental sustainability, on the one hand, and maintenance of fish growth, overall health/survival, while meeting fillet quality and consumer expectations, on the other [15,16,17]. Furthermore, “the icing on the cake” would be the development of aquafeeds that, besides satisfying the previous features, present the added value of exhibiting functional properties. The development of functional feeds, characterized as having physiological effects in fish beyond those of nutrient effects (e.g., improvement of stress tolerance, disease resistance, and health performance) [18], has, in past years, increasingly attracted the attention of research in food sciences applied to aquaculture [19]. Functional ingredients/nutrients in aquafeeds can be designed to achieve a plethora of expected benefits, but the strengthening of antioxidant defenses and DNA integrity, due to their pivotal roles determining plentiful upstream functions, stands among those that could result in greater physiological improvements and therefore have higher potential to generate outcomes in this productive activity.

The antioxidant defense system is essential to maintain cell homeostasis. When it fails or is overwhelmed, oxidative stress products (such as reactive oxygen species; ROS) can damage cell constituents such as lipids, proteins, and DNA. Fish can adapt to ROS overgeneration by increasing antioxidant defenses; however, the amount of ROS that can be neutralized is limited, which may disturb the redox state [20,21,22], impairing critical functions at higher levels. It has been demonstrated that antioxidant shielding in fish can be strengthened by dietary approaches, through supplementation with natural or artificial ingredients such as selenium [23], oligosaccharides of plant origin and phytogenic feed additives [24], carotenoids [25], and diludine [26].

Genome integrity and stability have central importance to organisms’ health, fitness, and ultimately survival. Intensive aquaculture practices (e.g., health management through the use of anesthetics, antiparasitics, and antibiotics) and the manipulation of rearing conditions (e.g., stressing temperature and high stocking density) pose recurring challenges to the DNA integrity of farmed fish [27]. Thus, the improvement of basal levels of DNA integrity (translating an efficacy to neutralize endogenous sources of damage), or the enhanced capacity to cope with the exogenous threats previously mentioned, play a key role in the balance between health and disease, in the proper functioning of metabolic pathways, and thereby on the general status and growth performance of fish. As stated above for oxidative status, genome integrity can be modulated, including in fish, by dietary factors, which was clearly demonstrated, for instance, with supplementation with vitamin C [28] and marine macroalgae [29]. Nevertheless, to the authors’ knowledge, the incorporation of halophytes in fish feeds as a factor of genoprotection was never investigated.

The European seabass (*Dicentrarchus labrax*), from the Moronidae family, was the first species of non-salmonid marine fish to be produced in Europe, being currently one of the most important species commercially exploited in the Mediterranean area (e.g., Greece, Turkey, Italy, Spain, Croatia, and Egypt) [30]. In 2020, global production of farmed *D. labrax* reached 243.9 thousand tons, its highest value ever [1]. Its predatory nature and inherent dietary requirements make the inclusion of plant-based ingredients more challenging. Nevertheless, the partial replacement of fish meal by plant protein sources in the diet of *D. labrax* has shown to be viable [30], though requiring a careful evaluation of the adequate percentage [31]. Concerning the development of functional feeds, in the last decades, different ingredients have been tested on *D. labrax*. For instance, the introduction of yeast as a complementary ingredient showed no negative effects on growth and improved feed efficiency [32]. Medicinal plants such as *Yucca schidigera* improved hematological parameters and immunological responses [33], while the introduction of prebiotics acted as immunostimulants through the increase of phagocytic activity [34]. It was also suggested that dietary supplementation with a macro- and microalgae blend may improve the intestinal health of *D. labrax* [35]. Recently, a study investigated the use of alternative feed resources from insects, crustacea, or microalgae biomass as functional supplements in a diet for juvenile *D. labrax* totally deprived of fish meal [36]. The results pointed out that supplementation with insect and crayfish meals improve fish growth and gut health, while the use of microalgae still requires some refinement.

Therefore, having as a backdrop the importance of *D. labrax* production in the European aquaculture sector and the promising attributes of halophytes enunciated above, the present work was designed to assess the functional properties of *S. ramosissima* when incorporated in *D. labrax* feed, coupled with an evaluation of the impact on growth performance. Subsequently, the specific goals considered were: (i) to evaluate the protection promoted by *S. ramossima* specifically towards the improvement of DNA and chromosomal integrity (in blood) and antioxidant system strengthening (in the liver, gills, and blood); (ii) to ensure that *S. ramossima* supplementation does not present adverse effects to seabass, translated into health impairments that may affect fish growth and welfare.

Ultimately, the pursuit of the previous goals will contribute to shedding light on the potential of halophytes as an emerging feed ingredient, towards the improvement of aquaculture practices and the valorization of low-value plant-based material.

## 2. Materials and Methods

### 2.1. Experimental Diets

*Salicornia ramosissima* biomass (21.5 kg) was collected in Praia da Areia Branca, Torreira (40°46′22.1″ N 8°39′29.5″ W; Portugal), in May 2019. Plant portions not used for human consumption (i.e., the whole plant, except the green tips and the roots) were isolated, and dried in Riasearch Lda. facilities (Murtosa, Portugal), in mesh mats that allowed air circulation, to prevent any degradation promoted by humidity. The resulting 6 kg were sent to Sparos Lda. (Olhão, Portugal) for feed formulation and manufacture, by extrusion (pellet size: 1.2 and 2.0 mm), after proper grinding (below 400 µm, as all the other ingredients). A control diet (C) was formulated to meet the nutritional requirements of juvenile *D. labrax*. Three isoproteic (wheat gluten levels were slightly adjusted to ensure protein levels remained similar between diets), isolipidic, and isoenergetic diets were produced, differing in the ingredient formulation from diet C only by replacing wheat meal with *S. ramosissima* whole-plant biomass, incorporated at 2.5%, 5%, and 10% of feed weight (S2.5, S5, and S10, respectively).

The main ingredients and analyzed proximate composition of the experimental diets are detailed in Table 1 and Table 2, respectively.

### 2.2. Rearing System, Feeding Trial, and Fish Sampling

Juveniles of European seabass (*D. labrax*), with a mean initial weight of 7.3 ± 0.1 g, were obtained from Sonríonansa, S.L. hatchery (Cantabria, Spain) and allocated in Riasearch Lda. facilities (Murtosa, Portugal). The trial, started in July 2020, consisted of assessing the growth performance and health condition (cytogenetic and biochemical evaluations) of *D. labrax* after the inclusion of different percentages of *S. ramosissima* in the feed. For that, fish were divided into 12 tanks (cylindrical fiberglass tanks of 350 L, each containing 200 L of water and 80 fish), corresponding to three tanks per condition/diet and a closed saltwater recirculation system (RAS) with 40 m^3^ (with a water renewal of 200 L per hour). During the experimental period, water parameters were measured once a day using commercial probes and maintained as follows: temperature at 21.6 ± 0.2 °C, dissolved oxygen at 6.4 ± 0.6 mg L^−1^, salinity at 18.2 ± 0.2, pH at 7.5 ± 0.2, and nitrogen compounds below 0.1 mg L^−1^.

A feeding trial with a total duration of 2 months, and an intermediate sampling after 1 month were performed, involving the diets described above (see Section 2.1). Fish were kept under a 14 h light:10 h dark photoperiod and fed by hand until visual satiety in three daily meals.

For the assessment of growth performance, body weight was determined at the end of the experimental period for all fish. The occurrence of mortality was monitored daily, and survival was expressed as a percentage and calculated as: S = (Sf/Si) × 100, where Si and Sf correspond to the initial and final number of individuals in the tanks, respectively.

At each sampling moment, for cytogenetic and biochemical evaluations, five fish were randomly collected from each thank (15 fish per condition/diet). Fish were anesthetized with 200 mg L^−1^ tricaine methanesulfonate (MS-222; buffered with NaHCO_3_) for approximately 10 min, and blood was drawn from the posterior cardinal vein using heparinized (27 mg mL^−1^ heparin) glass Pasteur pipettes and collected in 2 mL microtubes. Thus, one microtube, containing 0.002 mL of blood diluted in 1 mL of chilled PBS (pH = 7.4; 0.01 M), constituted the cell suspension for comet assay, and the other, with the remaining blood volume, was assigned for antioxidant analysis. Aliquots for comet assay were kept cold up to further procedures, while the aliquots for antioxidants determination were immediately frozen in liquid nitrogen. Blood smears were immediately prepared for erythrocytic nuclear abnormalities (ENA) assay and erythrocyte maturity index (EMI). Immediately after blood sampling, the fish were sacrificed by cervical transection. The gills and liver were removed and frozen in liquid nitrogen for later determination of oxidative stress endpoints.

### 2.3. Biochemical and Cytogenetic Evaluations

#### 2.3.1. Antioxidant System Status and Peroxidative Damage

All measurements were carried out in a SpectraMax 190 microplate reader, at 25 °C. Tissue samples were homogenized using a Potter–Elvehjem homogenizer, in chilled phosphate buffer (0.1 M, pH 7.4) in a 1:11 (blood and liver) and 1:6 (gills) ratio [tissue mass (mg): buffer volume (mL)]. The resulting homogenate of each tissue was then divided into two aliquots, for lipid peroxidation (LPO) measurement and post-mitochondrial supernatant (PMS) preparation. The PMS preparation was obtained by centrifugation in a refrigerated centrifuge (Eppendorf 5415R) at 13,400× *g* for 20 min at 4 °C. Aliquots of PMS were then divided into microtubes and stored at −80 °C until further antioxidant analyses. The activities of superoxide dismutase (SOD), catalase (CAT), glutathione peroxidase (GPx), and glutathione transferase (GST), as well as total glutathione (GSHt) content were measure in all the tissues addressed, while glutathione reductase (GR) activity was assessed only in the gills and liver (blood showed no measurable levels of this enzymatic activity).

SOD activity was assayed in PMS (at 25 °C) with a Ransod kit (Randox Laboratories Ltd., Crumlin, UK). The method employs xanthine and xanthine oxidase to generate superoxide radicals, which react with 2-(4-iodo-phenyl)-3-(4-nitrophenol)-5-phenyltetrazolium chloride (INT) to form a red formazan dye determined at 505 nm. SOD activity was then measured by the degree of inhibition of this reaction, considering that one unit of SOD causes a 50% inhibition of the rate of reduction of INT, under the conditions of the assay. The results were expressed as the SOD unit’s mg protein^−1^.

CAT activity was assayed in PMS (at 25 °C) by the method of [37], with slight modifications. Briefly, the assay mixture consisted of 0.190 mL phosphate buffer (0.05 M, pH 7.0) with hydrogen peroxide (H_2_O_2_; 0.010 M) and 0.010 mL of PMS, in a final volume of 0.2 mL. Change in absorbance was measured in appropriated UV transparent microplates (UV-Star^®^ flat-bottom microplates, Greiner Bio-One GmbH, Germany), recorded at 240 nm, and CAT activity was calculated in terms of μmol H_2_O_2_ consumed min^−1^ mg^−1^ protein, using a molar extinction coefficient (ε) of 43.5 M^−1^ cm^−1^.

GPx activity was determined in PMS (at 25 °C) according to the method described by [38] and modified by [39]. The assay mixture consisted of 0.09 mL phosphate buffer (0.05 M, pH 7.0), 0.03 mL ethylenediaminetetraacetic acid (EDTA; 0.010 M), 0.03 mL sodium azide (0.010 M), 0.03 mL glutathione reductase (2.4 U mL^−1^), 0.03 mL reduced glutathione (GSH; 0.010 M), 0.03 mL nicotinamide adenine dinucleotide phosphate-oxidase (NADPH; 0.0015 M), 0.03 mL H2O2 (0.0025 M), and 0.03 mL of PMS in a total volume of 0.3 mL. Oxidation of NADPH to NADP^+^ was recorded at 340 nm, and GPx activity was calculated in terms of nmol NADPH oxidized min^−1^ mg protein^−1^ using a ε of 6.22 × 10^3^ M^−1^ cm^−1^.

GST activity was determined in PMS (at 25 °C) with CDNB (1-chloro-2,4-dinitrobenzene) as a substrate, according to the method of [40]. The assay mixture consisted of 0.1 mL of PMS and 0.17 mL of phosphate buffer (0.2 M, pH 7.9) and GSH (0.0018 M). The reaction was initiated by addition of 0.03 mL of CDNB (0.01 M), and the increase in absorbance was recorded at 340 nm. The enzyme activity was calculated as nmol CDNB conjugate formed min^−1^ mg^−1^ protein (ε = 9.6 mM^−1^ cm^−1^).

GR activity was measured according to [41] in PMS (at 25 °C), with minor modifications. Briefly, the assay mixture contained 0.050 mL of PMS fraction and 0.250 mL of reaction medium consisting of phosphate buffer (0.05 M, pH 7.0), NADPH (0.0002 M), glutathione disulfide (GSSG; 0.001 M) and diethylenetriaminepentaacetic acid (DTPA; 0.0005 M). The activity was determined by measuring the oxidation of NADPH at 340 nm and calculated as nmol NADPH oxidized min^−1^ mg protein^−1^, using a ε of 6.22 × 10^3^ M^−1^ cm^−1^.

For GSHt content determination, PMS was precipitated with trichloroacetic acid (TCA 12%) for 1 h and then centrifuged at 12,000× *g* for 5 min at 4 °C. GSHt was determined (in deproteinated PMS) by adopting the enzymatic recycling method with GR excess, whereby the sulfhydryl group of GSH reacts with 5,5-dithiobis-(2-nitrobenzoic acid) (DTNB; Ellman’s reagent) and produces a yellow-colored 5-thio-2-ni-trobenzoic acid (TNB) [42,43]. The rate of TNB production is directly proportional to this recycling reaction, which is in turn directly proportional to the GSH concentration in the sample. The assay mixture consisted of 0.2 mL sodium phosphate buffer (0.143 M, pH 8), EDTA (0.0063 M), DTNB (0.001 M), and NADPH (0.00034 M), added to 0.04 mL of deproteinated PMS. The reaction was initiated with 0.04 mL of GR (8.5 UmL^−1^). The formation of TNB was measured at 415 nm. It should be noted that GSSG is converted to GSH by GR in this system, which consequently measures total GSH. The results were expressed as nmol TNB formed min^−1^ mg protein^−1^ (ε = 14.1 × 10^3^ M^−1^ cm^−1^).

As an estimation of LPO, the quantification of thiobarbituric acid reactive substances (TBARS) was carried out in the previously prepared lysate according to the procedure of [44,45], adapted by [46,47]. Briefly, 0.005 mL of butylatedhydroxytoluene (BHT; 4% in methanol) and 0.045 mL of potassium phosphate buffer (0.05 M, pH 7.4) were added to 0.075 mL of lysate and mixed well to prevent oxidation. To 0.05 mL of this mixture, 0.25 mL of TCA (12%) were added and vortexed, and then, 0.225 mL of Tris–HCl (0.06 M) and DTPA (0.0001 M) (pH 7.4) and 0.25 mL of thiobarbituric acid (TBA; 0.73%) were added. This mixture was heated for 1 h in a water bath set at 100 °C and then cooled to room temperature and centrifuged (Eppendorf 5415R) at 15,700× *g* for 5 min. The absorbance of each sample supernatant was measured at 535 nm. LPO was expressed in nmol of TBARS-formed mg tissue^−1^ using a ε of 1.56 × 10^5^ M^−1^ cm^−1^.

#### 2.3.2. DNA and Chromosomal Integrity

The alkaline comet assay was performed based on the method described by [48], with slight modifications according to [49,50]. All slides were freshly prepared. Briefly, 20 µL of cell suspension (using the whole blood previously diluted in PBS) was resuspended in 70 µL of low melting point agarose (1%; dissolved in PBS). Twelve drops (gels) with 6 µL of cell suspension were placed on a pre-coated glass slide with 1% of normal melting-point agarose (1%; dissolved in distilled water), as two rows of six gels (six groups of two replicates), without coverslips, containing approximately 1.5 × 10^3^ cells/gel. Gels were kept for 5 min at 4 °C to let agarose polymerise and then immersed in a lysis solution (2.5 M NaCl, 0.1 M EDTA, 10 mM Tris, 1% Triton X-100, pH 10) for 1 h. Thereafter, slides were gently placed in a horizontal electrophoresis tank filled with fresh electrophoresis solution (0.3 M NaOH, 1 mM EDTA, pH 13), for alkaline treatment. DNA was then allowed to unwind for 20 min. Electrophoresis was performed under 1.04 V cm^−1^ for 15 min. DNA unwinding and electrophoresis (as well as the preceding lysis) were carried out in the dark, at 4 °C. Once finished, the electrophoresis slides were washed in PBS (10 min), distilled water (10 min), and then gels were fixed for 10 min in absolute ethanol. Slides were stained with ethidium bromide (20 g L^−1^) and analyzed on a Leica DM2000 fluorescence microscope (×100 magnification). Images were captured with a Leica DFC7000 T camera. Tail DNA % was determined with OpenComet software [51] with a minimum of 100 nucleoids per sample. This metric for the comet assay is the most accepted in use for determining DNA breaks [52,53,54]. Automatically generated scores corresponding to multiple nuclei, non-cellular debris, or obstructions were manually removed from the analysis.

The erythrocytic nuclear abnormalities (ENA) assay was carried out in mature peripheral erythrocytes, according to the procedure of [55]. Briefly, one blood smear per animal was fixed with methanol for 10 min and stained with Giemsa (5%) for 30 min. Slides were coded and scored blind. From each smear (one slide per fish), 1000 erythrocytes were scored, under 1000× magnification (microscope Olympus BX50), to evaluate the relative frequency of the following nuclear lesions: kidney shaped nuclei (K), lobed nuclei (L), segmented nuclei (S), vacuolated nuclei (V), and micronuclei (MN). The results were expressed as the sum of frequencies for all the categories observed (K + L + S + V + MN).

#### 2.3.3. Erythrocyte Population Dynamics

**Figure 1:**
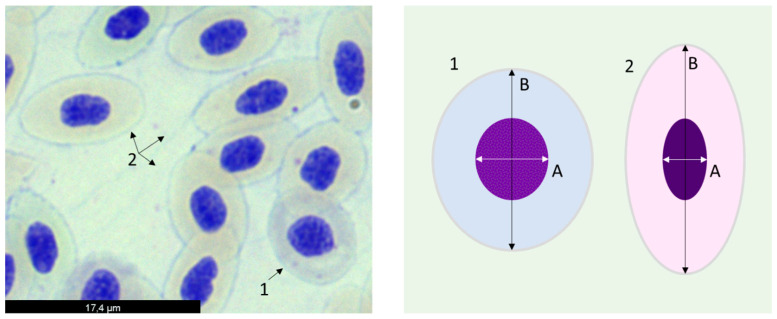
**Peripheral erythrocytes of *Dicentrarchus labrax*.** (**Left**): blood smear (Giemsa stain) showing erythrocytes in different stages of maturation. (**Right**): representation of erythrocytes (with nuclear normal shape) elucidating measurements performed for the calculation of the erythrocyte maturity index (EMI), viz. minor axis of the nucleus (A) and major axis of the cell (B). In both images, erythrocytes in earlier (1) and later (2) maturity stages are represented.

The erythrocyte population dynamics were assessed by the calculation of the erythrocyte maturity index (EMI), determined according to [56], with the modifications proposed by [57]. Briefly, 10 microscopic fields were randomly selected per slide (one slide per fish; the same slides used for the ENA assay) and photographed under 400× magnification (microscope Olympus BXB50). Then, in each microscopic field, 25 random cells were analyzed with ImageJ software, measuring the minor axis of the nucleus and the major axis of the cell (A and B, respectively; see Figure 1).

The EMI was calculated for each cell by dividing A by B values, to a total of 250 cells. From the values of the ratio, cells were then categorized into one of the 10 maturity classes: [0.0 ≤ class 1 < 0.1]; [0.1 ≤ class 2 < 0.2]; [0.2 ≤ class 3 < 0.3]; [0.3 ≤ class 4 < 0.4]; [0.4 ≤ class 5 < 0.5]; [0.5 ≤ class 6 < 0.6]; [0.6 ≤ class 7 < 0.7]; [0.7 ≤ class 8 < 0.8]; [0.8 ≤ class 9 < 0.9]; and [0.9 ≤ class 10 ≤ 1], where class 1 represented erythrocytes with the higher maturity level and class 10 corresponded to cells with lower maturity status. Finally, average values for the frequency (%) of cells observed in each maturity class were represented for each experimental group.

### 2.4. Statistical Analyses

The statistical analysis was conducted on the free trial of Statistica 10.0 software (StatSoft, Inc., Street Tulsa, OK, USA), considering each sampling moment individually. The normality and homogeneity of variances were confirmed by Shapiro–Wilk’s W and Brown–Forsythe (HOV) tests, respectively, in order to meet the required statistical demands.

Differences in growth performance and survival between dietary treatments were evaluated using one-way ANOVA, followed by Tukey HSD multiple comparison tests. Kruskal–Wallis one-way analysis of variance tests, followed by Wilcoxon pairwise comparison tests were used when data did not comply with the one-way ANOVA’s assumptions.

For biochemical and cytogenetic data, one-way ANOVA followed by the post hoc Dunnett test were used to compare test-diet groups with the corresponding control. When the parametric demands failed, the statistical nonparametric Kruskal–Wallis test was performed. When the data did not fulfil the assumptions for normality, a non-parametric test was executed (Kruskal–Wallis ANOVA) and, when detected differences, a post-hoc Dunn’s test was applied.

In results expressed as percentages, an arcsine transformation was performed prior to any statistical test: T = ASIN (SQRT (value/100)), and then tested for normality (Shapiro–Wilk test and graphical analysis) and homogeneity of variances (Levene’s test).

In all the analyses, differences between means were considered significant when *p* < 0.05 [58].

## 3. Results

### 3.1. Growth Performance

Fish grew properly, and no significant differences between dietary treatments were found at the end of the feeding trial (month 2), in body weight as well as in survival values. Thus, final body weights (g) were 43.70 ± 0.32, 43.30 ± 1.28, 43.60 ± 0.98 and 43.50 ± 0.95, respectively for C, S2.5, S5, and S10. Survival values (%) were 94.60 ± 4.10, 97.10 ± 1.60, 97.90 ± 1.20, and 95.80 ± 1.60, respectively for C, S2.5, S5, and S10.

### 3.2. Antioxidant Defenses vs. Oxidative Damage

**Figure 2:**
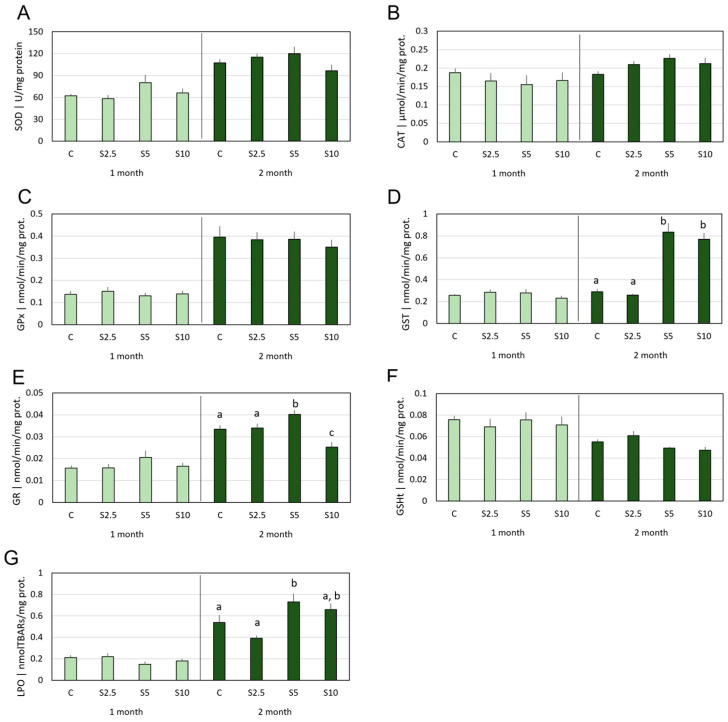
**Mean values of oxidative stress parameters in the liver**, namely (**A**) superoxide dismutase (SOD), (**B**) catalase (CAT), (**C**) glutathione peroxidase (GPx), (**D**) glutathione-S-transferase (GST), (**E**) glutathione reductase (GR) activities, and (**F**) total glutathione content (GSHt), as well as (**G**) lipid peroxidation (LPO), following 1 (light green) and 2 months (dark green) of dietary supplementation. Experimental groups concern: control (C), fed with standard feed, and *Salicornia*-supplemented diets (S2.5, S5, and S10, corresponding to 2.5%, 5%, and 10% supplementation, respectively). Bars represent standard errors. Different letters correspond to statistically significant differences (*p* < 0.05), within the same trial duration (lowercase letters for 2 months).

In the first month of the feeding trial, no variations in antioxidants nor lipid peroxidation were observed in the livers of the fish. Nevertheless, following 2 months of dietary supplementation, the S5 group showed significantly increased activities of GST and GR (Figure 2D,E), as well as an elevation of LPO levels (Figure 2G), when compared with the C group. At the same point in time, the S10 group also showed a GST activity increase, but occurring concomitantly with a GR activity decrease.

**Figure 3:**
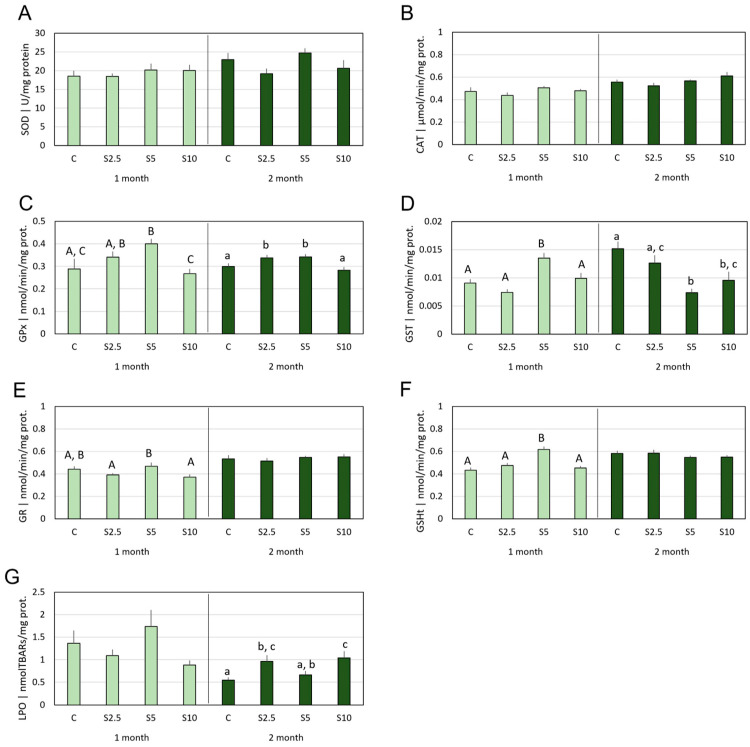
**Mean values of oxidative stress parameters in the gills**, namely (**A**) superoxide dismutase (SOD), (**B**) catalase (CAT), (**C**) glutathione peroxidase (GPx), (**D**) glutathione-S-transferase (GST) activities, (**E**) glutathione reductase, and (**F**) total glutathione content (GSHt), as well as (**G**) lipid peroxidation (LPO), following 1 (light green) and 2 months (dark green) of dietary supplementation. Experimental groups concern: control (C), fed with standard feed, and *Salicornia*-supplemented diets (S2.5, S5, and S10, corresponding to 2.5%, 5%, and 10% supplementation, respectively). Bars represent standard errors. Different letters correspond to statistically significant differences (*p* < 0.05), within the same trial duration (capital letters for 1 month; lowercase letters for 2 months).

Concerning gills, in the first month, significant alterations in relation to group C were restricted to the S5 dietary group. Hence, this group depicted an increase of GPx and GST activities (Figure 2C and Figure 3D), as well as GSHt content (Figure 3F). Simultaneously, for the same group, no alterations were detected in SOD and CAT activities (Figure 3A,B), nor LPO (Figure 3G). A different response pattern was perceived in month 2, when a significant increase of GPx activity was measured in the S2.5 and S5 groups (Figure 3C), and a GST activity decrease in S5 and S10 (Figure 3D), while SOD and CAT activities remained unaltered (Figure 3A,B). Lipid peroxidation showed significant increases in the S2.5 and S10 groups (Figure 3G).

**Figure 4:**
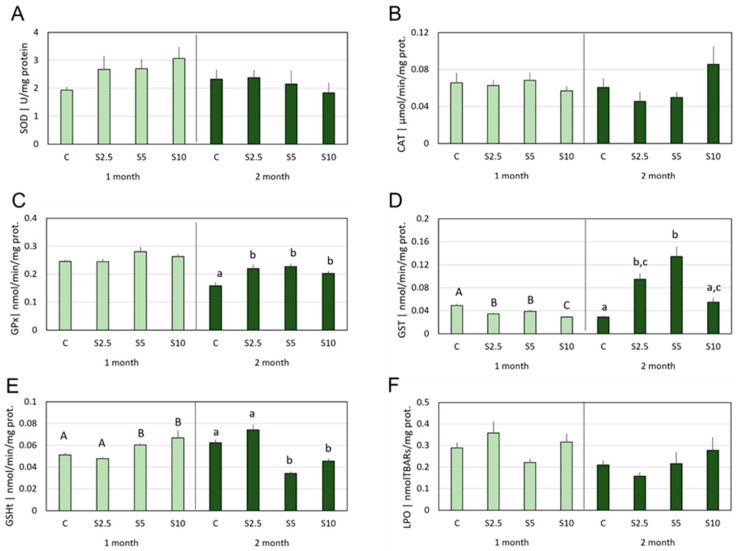
**Mean values of oxidative stress parameters in the blood**, namely (**A**) superoxide dismutase (SOD), (**B**) catalase (CAT), (**C**) glutathione peroxidase (GPx), (**D**) glutathione-S-transferase (GST) activities, and (**E**) total glutathione content (GSHt), as well as (**F**) lipid peroxidation (LPO), following 1 (light green) and 2 months (dark green) of dietary supplementation. Experimental groups concern: control (C), fed with standard feed, and *Salicornia*-supplemented diets (S2.5, S5, and S10, corresponding to 2.5%, 5%, and 10% supplementation, respectively). Bars represent standard errors. Different letters correspond to statistically significant differences (*p* < 0.05), within the same trial duration (capital letters for 1 month; lowercase letters for 2 months).

In blood, as reported for the other tissues, no significant differences were detected between the experimental groups (in both sampling moments) for SOD and CAT activities (Figure 4A,B).

The other antioxidants measured displayed distinct response patterns in the two trial lengths. Hence, regarding GPx activity, significant differences were only detected in the second month, with all the supplemented groups (S2.5, S5, and S10), showing higher levels in comparison with the control group, but no differences among them (Figure 4C).

Regarding GST activity (Figure 4D), after 1 month, all the supplemented groups displayed lower levels when compared to the respective control, with the highest supplementation group (S10) also showing significantly lower activity in comparison with the other supplemented groups (S2.5 and S5). Differently, after 2 months, groups S2.5 and S5 showed significantly elevated GST activity in comparison to the control, with the highest level reported for S5 (also significantly higher than S10). Concerning GSHt, time-related profiles were also found (Figure 4E), since, in the first month, groups S5 and S10 revealed significantly higher contents in comparison with the control group (as well as with S2.5), while, after 2 months, the same groups displayed significantly lower content in comparison with the control group (as well as with S2.5). In terms of LPO, no significant alterations were detected either in the first or in the second month (Figure 4F).

### 3.3. DNA and Chromosomal Integrity

**Figure 5:**
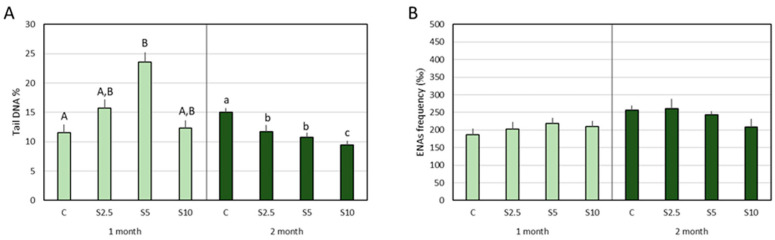
**DNA and chromosomal damage in blood cells of the European seabass**. DNA strand breaks measured by the comet assay (**A**), and expressed as percentages of DNA in the tail, and erythrocytic nuclear abnormalities frequency (‰) (**B**), following 1 (light green) and 2 months (dark green) of dietary supplementation. Experimental groups concern: control (C), fed with standard feed, and *Salicornia*-supplemented diets (S2.5, S5, and S10, corresponding to 2.5%, 5%, and 10% supplementation, respectively). Bars represent standard errors. Different letters correspond to statistically significant differences (*p* < 0.05), within the same trial duration (capital letters for 1 month; lowercase letters for 2 months).

Significant differences in terms of tail DNA% were detected in both months but translating contrasting variation profiles. In the first month, the S5 group revealed significantly higher DNA damage when compared to the control group. In the second month, all the supplemented groups (S2.5, S5, and S10) showed significantly lower values of DNA damage when compared to the control group, with the lowest percentage (highest DNA integrity) measured for S10 (Figure 5A).

During the two months of the trial, no significant differences were detected for the ENA frequency (Figure 5B).

### 3.4. Erythrocyte Population Dynamics (EMI Assay)

**Figure 6:**
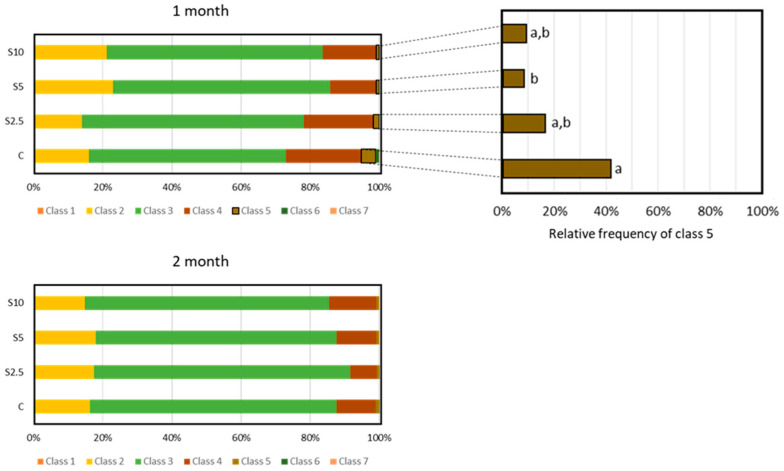
**Representation of frequency (%) of classes 1 to 7 of the erythrocyte maturity index (EMI)** evaluated in peripheral erythrocytes, following 1 (**top-left**) and 2 months (**bottom**) of dietary supplementation, and class 5 (**top-right**) in the first month. Experimental groups concern: control (C), fed with standard feed, and *Salicornia*-supplemented diets (S2.5, S5, and S10, corresponding to 2.5%, 5%, and 10% supplementation, respectively). Bars represent standard errors. Different lowercase letters correspond to statistically significant differences (*p* < 0.05).

The cell frequency for each maturity class is depicted in Figure 6 (classes 1 to 7; no cells in classes 8 to 10 were observed). The most representative class in both sampling moments, regardless of the experimental group, was class 3.

Significant differences between the groups were detected only for class 5 and in the first month of the experimental trial. The group fed with 5% *S. ramosissima* (S5) revealed a lower frequency of these cells when compared to the control group (Figure 6).

## 4. Discussion

In past decades, developments in fish nutrition towards the identification of functional feeds and a thorough understanding of underlying mechanisms have been made; however, research in this area has been mainly focused on animal growth, feed intake, fillets’ physicochemical and sensory properties, and histological analysis of the digestive apparatus and gut microbiome [59]. In more recent years, studies of DNA and chromosomal integrity, as well antioxidant system modulation, have been performed in fish [60,61], highlighting the potential advances for the aquaculture sector resulting from the scientific knowledge to be developed in this context.

About 2000 plants are known worldwide to possess some salinity tolerance, but only a few species have been investigated for their antioxidant capacities [3], thus adding a greater scientific interest to the current study.

In the light of the statement “a feed is only as good as its ingredients” [62], and considering the phytochemical profiling of *Salicornia* spp. described in the literature [10,11,12] and the beneficial health properties already demonstrated in other contexts, the potential of these halophytes as functional ingredients of fish feeds bursts forth as a plausible hypothesis. Nevertheless, this has never been addressed, including in the context of carnivorous species like the European seabass (*Dicentrarchus labrax*), which points out the novelty of the present investigation.

Though this research was focused on the functionality of dietary supplementation with *S. ramosissima* biomass, some considerations on aquafeed environmental sustainability can still be made. Functional additives are supposed to be incorporated at low concentrations, but it must be highlighted that the highest inclusion level currently tested (10%), replacing wheat meal, represents a saving in cereal use of 63%, compared with a regular feed, which is likely to have impacts beyond functionality (such as in terms of economic and environmental sustainability). This should be just one aspect of a larger goal of improving the environmental performance of global aquaculture [63] that also includes the reduction the Fish In: Fish Out (FIFO) ratio [64], and has been increasingly placed on the agenda of researchers, feed and fish meal/oil producers, and regulation and advisory agencies (e.g., the European Aquaculture Advisory Council).

### 4.1. Growth Performance of European Seabass following S. ramosissima Dietary Supplementation

As previously stated, from the point of view of farmers and feed manufacturers, the primary condition for a novel plant-based raw material to be pondered as a functional ingredient for fish feeds (in addition to costs) is the maintenance of zootechnical performance (e.g., survival, growth, feed palatability, and feeding efficiency) [15,16,17]. In this direction, current data indicate that *S. ramosissima* biomass can be included in diets for juvenile *D. labrax*, at up to 10% of their composition, with no detrimental effects to growth performance and survival. This agrees with previous works on *D. labrax* nutrition, which demonstrated that macroalgae- [65] and plant-based [66] meals do not negatively affect growth.

In the present work, a percentage of wheat meal was replaced by halophyte biomass in *D. labrax* feed. Wheat meal has been introduced in fish feed formulations since it is a good source of energy due to its high carbohydrate content and dietary fiber, and can also complement protein sources [67]. Hence, we also demonstrated the correctness of the decision regarding the partial replacement of wheat, instead of another ingredient, in regular *D. labrax* feeds.

Hence, once the key factor regarding growth performance and survival was guaranteed, the conditions were met to move on to the next step, exploring the potential functional properties of *S. ramosissima*.

### 4.2. Incorporation of S. ramosissima in European Seabass Feed and Modulation of the Antioxidant System and Oxidative Status

*Salicornia* spp. are known to be a good source of antioxidants [9,10,11,68]. For instance, it has been demonstrated that *S. herbacea* could be a useful antioxidant due to its phenolic (salycilic, p-coumaric, ferulic, caffeic acids) and flavonoid (quercetin, acacetin) compounds [68]. Phenolic compounds are known to have direct antioxidant actions due to the presence of hydroxyl groups, which function as hydrogen donors [69]. Concerning specifically *S. ramosissima*, it was reported that its antioxidant capacity may be related to compounds such as vitamins (e.g., β-carotene and α-tocopherol) [10], flavonoids, phenolics, and hydroxycinnamic acid derivatives [11,70]. Therefore, the assumptions are met to put forward the hypothesis that dietary supplementation with *S. ramosissima* can improve the oxidative status of *D. labrax*.

The antioxidant system is essential for maintaining cellular and tissue integrity, as fish, like all aerobic organisms, are exposed to pro-oxidant pressures due to normal (endogenous) metabolic processes and exogenous factors [71,72]. It has been clearly established for fish, including for *D. labrax* [73], that antioxidant responses are tissue specific, showing differential expression of defense mechanisms in various organs/tissues due to their distinct functions, metabolic rates, and exposure to environmental stressors resulting from their anatomical position. Hence, though an integrated picture of the different tissues addressed is envisioned, the current results will first be discussed, respecting this specificity.

The liver is the central metabolic organ in fish [74], responsible for an array of functions, and the principal site of xenobiotic metabolism and detoxification of harmful substances immediately after their absorption from the gastrointestinal tract [74]. Therefore, this organ developed a robust antioxidant defense system to protect its own cellular structures and maintain overall health. This robustness of the hepatic antioxidant system (which also includes a variety of non-enzymatic antioxidants not measured in the present study, like vitamin C, vitamin E, and various metal-binding proteins) may justify the “immobility” (stability) of the antioxidants addressed in the liver after the first month of supplementation with *S. ramosissima*. Unlike gills and blood, the liver took 2 months for the GSH-associated enzymes (GST and GR) to respond, which, in coherence, only occurred in the higher levels of supplementation (5% and 10%), with the lower level (2.5%) remaining unresponsive. Moreover, it seems that the incorporation of 5% of *S. ramosissima* acted as a challenge to the hepatic antioxidant system, which, in addition to increasing GST and GR activities, allowed the rise of LPO levels. A preliminary interpretation of these results would point to the existence of a certain level of toxicity associated with the halophyte ingestion. However, a more comprehensive discussion will be developed below, pointing out another interpretation.

Antioxidant defenses in organs that interface with the external medium and are involved in gas exchange, such as fish gills, are reinforced as they are highly exposed to environmental factors that can lead to the production of ROS. Thus, the branchial GSH-related defense subsystem was particularly responsive in fish fed with 5% of *S. ramosissima* for 1 month, displaying increased GPx, GST, and GR activities, as well as GSHt content. Interestingly, the gills of fish fed with the diets incorporating 2.5% and 10% remained unresponsive. The GSH-related defense subsystem plays a crucial role in protecting cells and organisms from oxidative stress and various toxic substances [75], and refers to a set of biological mechanisms and molecules centered around the tripeptide molecule glutathione (GSH), which, in addition to the elements analyzed here, further includes GSH biosynthesis and GSH transporters. It seems clear that, within relatively narrow limits, one month of *S. ramosissima* supplementation can upregulate GSH-related defenses in juvenile seabass. After 2 months of dietary supplementation, the modulation of these antioxidants followed a distinct profile, with a less distinguishable pattern (GPx induction in S2.5 and S5; GST decrease in S5 and S10), coupled with the emergence of lipid peroxidation in the S2.5 and S10 groups.

A solid discussion on these results is necessary here, considering the complexity of the underlying processes. There is not a universally agreed-upon specific level of lipid peroxidation increase that is considered toxicologically worrying to fish, because it can depend on several factors, including baseline levels, duration of exposure, tissue/organ specificities, and concurrent factors. Considering the interference of all these factors, there may be a dual interpretation for an increase of LPO; on the one hand, it can be a consequence of a failure/overwhelm of the antioxidant system, but on the other, it can signal a trigger for the activation of the antioxidant system. In some cases, exposure to moderate levels of ROS and lipid peroxidation can stimulate an adaptive response, making the cells more resilient to future oxidative pressures [61]. This interplay between lipid peroxidation and the antioxidant system is a vital part of an integrated response to oxidative stress and the efforts to maintain/restore cellular homeostasis and tissue integrity. Therefore, the most plausible explanation for the punctual elevations of LPO, observed following 2 months of dietary supplementation with *S. ramosissima*, is that some plant constituents generate an external stimulus for the liver (at 5%) and gill (at 2.5% and 10%) cells of seabass, with the goal of strengthening the antioxidant defense system.

It has been demonstrated that polyphenols, also known for their antioxidant effects, can also have a pro-oxidant action associated with their instability and easy oxidation, generating H_2_O_2_, quinones, and semiquinones, among other products [69]. These compounds can thus exert a mild stressful challenge that triggers a rapid response, leading to increased levels of endogenous antioxidants, such as GSH [69,76]. Hence, flavonoids, a subgroup of polyphenols occurring in *Salicornia* spp. [11,68,70], are plausible candidates to play that dual role.

Corroborating the previous interpretation, it should be highlighted that certain foods or isolated compounds that are usually described as antioxidants also display pro-oxidant actions in humans [77,78]. In addition, several studies reported pro-oxidant properties of certain phytocompounds, namely carotenoids [79], vitamin C [80], and polyphenols [81].

It is worth noting that the cogitated mild pro-oxidant action of *S. ramosissima* displayed a dose-dependence specificity for the organs in equation, i.e., it occurs at 5% supplementation for the liver and at 2.5% and 10% for the gills. Moreover, the relevance of the determinants mentioned above emerges as obvious, namely organ specificities, duration of exposure (response evident only for 2 months of supplementation), and concurrent factors, which can explain the non-linear relationship to the dose (likely following hormetic or J-shaped dose-response curves).

Concerning blood cell responses, a distinct pattern was observed in comparison to the liver and gills. The only similarities that could be invoked between the three tissues addressed are the unresponsiveness of SOD and CAT activities to the *S. ramosissima* supplementation and the occurrence of clearly different response patterns in the two trial lengths (1 vs. 2 months). Therefore, *S. ramosissima* was shown to modulate the GSH-related subsystem, translated into a GST activity decrease (all the dietary groups) and increased GSH content (S5 and S10) in the first month of supplementation, while in the second month, a GPx (all the dietary groups) and GST (S2.5 and S5) induction was observed.

It is noteworthy from these results that the GSH-related subsystem in blood cells can be up- or down-regulated by *S. ramosissima* supplementation. Both directions of this modulation seem to represent improvements of antioxidant status due to supplementation, though the decrease of GST activity (month 1) and GSHt content (month 2) can be particularly interesting considering the potential to favorably impact fish growth. This assertion relies on the contribution of an exogenous source (dietary supplementation) of nonenzymatic antioxidants, saving endogenous resources via a lower expression/synthesis of antioxidant/free radical scavenger molecules, as suggested by [60,82] for the mechanism underlying the benefits of a macroalgae-enriched fish feed. A fish study carried out by [83] involving dietary vitamins also lends support to this interpretation, configuring what was called the theory of sparing.

Specifically regarding blood GSHt in the first month, the increased contents displayed by groups S5 and S10 can rely on the concomitant decrease of GST activity. GSTs represent a multigene family of primarily soluble enzymes involved in the detoxification of electrophilic and genotoxic xenobiotics, as well as endogenous reactive intermediates produced during cellular oxidative stress like peroxidative products [84,85]. Hence, a decrease in GST activity means that less GSH is being used for detoxification processes, allowing more GSH to accumulate within the cells.

Overall, the present results are in conformity with [82,86], who stated that LPO increase cannot be predicted only on the basis of antioxidant depletion. Predicting peroxidative damage as a function of antioxidant responses can be challenging due to the complex, intricate, and multifaceted nature of the processes involved, depending on factors such as the involvement of multiple antioxidant sub-systems, interactions between antioxidants, time-dependent dynamics, and feedback mechanisms.

During the whole trial, and in all tissues addressed, there were no variations in the activity of two key enzymatic antioxidants—SOD and CAT—categorized as a first line of defense. Moreover, this unresponsiveness was registered frequently in concomitance with the modulation of the GSH-related antioxidant subsystem. Both lines of defense represent different and complementary mechanisms for combating oxidative stress. SOD and CAT primarily deal with the breakdown of superoxide radicals (O2•-) and hydrogen peroxide (H_2_O_2_), while the GSH-related subsystem focuses on neutralizing other ROS, such as lipid peroxides and organic hydroperoxides [72,87]. Thus, it may be hypothesized that *S. ramosissima* supplementation entails a specific form of (mild) oxidative challenge, shared by the three tissues studied, pointing out the ability of seabass to fine-tune its defenses to match the specific nature of the oxidative stress it encounters.

### 4.3. Incorporation of S. ramosissima in European Seabass Feed and DNA/Chromosomal Integrity

The antigenotoxic properties of natural edible products (e.g., fruit, green tea) have been frequently investigated, using human experimental approaches, aiming at the identification of functional foods [88,89,90]. Differently, in the context of aquafeed formulation, though the concept has been also valued, research is much less profuse. Promising results were obtained, for instance, with the introduction of a macroalgae mixture in feeds for *S. aurata*, demonstrating a genoprotection effect [60], including against aqua-medicines frequently adopted in aquaculture (the antibiotic oxytetracycline and the antiparasitic formalin) [27]. However, the genoprotective potential of *S. ramosissima* incorporated in fish feeds, to the authors’ knowledge, is a completely unexplored topic.

Antigenotoxic protection was investigated in the present study through two complementary endpoints (both focused on blood cells): (i) DNA breaks measured by the comet assay (tail DNA %), a type of damage that can be repaired, and (ii) ENA frequency, translating to a more severe and less transient type of genetic damage (clastogenic and/or aneugenic lesions).

The results showed that chromosomal integrity (measured by ENA frequency) was not affected by the dietary treatments, indicating that more subtle modulation processes are at play. In fact, comet assay data pointed out measurable alterations, reflecting (apparently) contrasting results for 1-month and 2-month supplementation. In the first month, a punctual elevation of DNA damage was registered for the group fed with 5% *S. ramosissima*. As discussed above for oxidative stress data, this effect should be interpreted cautiously, taking into consideration the fact that it resulted from a delicate balance between genotoxic and antigenotoxic pressures. In fact, it is well established that substances described as DNA integrity promoters depict a mild genotoxic action, which may be a path to trigger the antigenotoxic defenses (e.g., [91,92,93,94]). These complex mechanisms, representing “two sides of the same redox coin” [88], were partially decoded using in vitro approaches (human lymphocytes) and addressing the protective effects of green tea (*Camellia sinensis*) [88]. Thus, it was suggested that genoprotection by low-dose green tea could be due to the direct antioxidant protection of polyphenols, while genotoxicity is induced by H_2_O_2_ (generated by autoxidation of catechins).

The integrated analysis of the different results presently obtained provide support to the interpretation of the DNA integrity alteration registered for the S5 group (month 1) in light of the mechanisms enunciated above, thus translating as mild and temporary damage that works as a trigger to activate the DNA protective pathways. In this direction, it should be considered that: (i) no increased DNA breaks were observed, for the same trial duration, in fish fed with 10% of *S. ramosissima* (which would be expected if it were a real toxic action); (ii) no chromosomal damage nor lipid peroxidation were concomitantly observed (S5 in month 1); (iii) the effect was no longer noticeable after 2 months. Hence, what could be described as a sign of genotoxicity is better described as a pro-genoprotective action.

As a further reinforcement of this explanation, following 2 months, comet assay results demonstrated that *Salicornia*-enriched diets offer solid genoprotection to all the groups with *Salicornia* supplementation, with emphasis on the S10 group that showed the most significant improvement to DNA integrity.

Similar to what was described by [88], it can be hypothesized that the genoprotective and genotoxic (pro-genoprotective) actions may be mediated by different *S. ramosissima* constituents, with distinct toxicokinetics and toxidynamics in the fish body. Nevertheless, no support is currently found for H_2_O_2_ to play the role of genotoxicant, since the results regarding antioxidant modulation do not confirm that specific mechanism, since CAT activity was regularly unresponsive. Elucidation of these complex mechanisms requires further investigation.

Looking closely, the results obtained after 1 and 2 months of supplementation will be complementary rather than contrasting, as the protective potential expressed over the longer period will have an underlying and intermediate state of preparation.

DNA integrity is vital for any organism, in any context, but it assumes paramount importance in fish under intensive culture (involving, for instance, high-density populations, close confinement, controlled environments, variations in water quality, and exposure to aqua-medicines) for several reasons: (i) is essential for preserving genetic diversity within fish populations, also associated with the ability to cope with stressors, reducing the risk of stress-related illnesses; (ii) it is linked to the immune system’s ability to recognize and combat pathogens, thereby promoting a robust immune response; (iii) any genetic defects or mutations can compromise growth; (iv) it allows saving the energy that would be allocated to defense mechanisms related, for example, to DNA damage repair or replacement of damaged cells.

In addition to the previous aspects, centered on fish health, it is also relevant to point out that maintaining DNA integrity helps ensure that the final products meet safety and quality standards, as well countering some criticism related to animal welfare and ethical concerns.

In future works, it will be important to understand the effect of a diet enriched with *Salicornia* when fish are facing genotoxic and/or oxidative challenges arising from routine aquaculture practices.

To shed some light on the dynamics of the circulating erythrocyte population and possible interferences of the dietary backgrounds tested, the erythrocyte maturity index (EMI) was assessed (based on the analysis of nucleo-cytoplasmatic ratios). This index also complements the information provided by the ENA assay, elucidating the balance between erythropoiesis and cell removal, and thus about recovery pathways [27] (the fate of DNA/chromosomal lesions will depend upon replacement of damaged cells) [95]. The dynamics can be altered, for instance, through interference with hemoglobin production, assembly, or stability, as well as with the cell cycle and maturation of precursor cells [96].

Current EMI results demonstrated that, in general, the dietary backgrounds tested did not affect *D. labrax* erythrocyte dynamics, which also means that the genomic integrity assessment was not affected by fluctuations in the erythrocyte dynamics/lifespan. A single alteration was pointed out, which concerned a frequency reduction of cell in class 5 (a stage tending to be immature) for the S5 group at 1 month. The sporadic and transient nature of this fluctuation reduces its significance. However, it can be an indication that erythrocytes from seabass fed with 5% *Salicornia* had a slightly higher lifespan, which could reflect an improvement of blood cells’ condition due to halophyte ingestion. Moreover, the benefit of avoiding energy expenditure in cell rejuvenation is implicit.

## 5. Conclusions

Overall, the outputs of the present study, addressing the potential of the incorporation of the halophyte *S. ramosissima* in the diet of the European seabass (*D. labrax*), allow the following conclusions:

(i) The inclusion of whole-plant biomass up to 10%, for 2 months, replacing wheat meal, showed no impairments on growth performance, pointing out that the use of *S. ramosissima* routinely as a feed ingredient can be considered, though its scalability and economic viability need to be evaluated.
(ii) *S. ramosissima* was shown to affect the oxidative status of *D. labrax*, namely modulating the GSH-related defense subsystem in a tissue-specific manner and with higher acuity in the second month of feeding. Though an antioxidant improvement was apparent, it followed multiple and complex mechanisms, which also included a mild pro-oxidant that, expectantly, triggered beneficial adaptations of the endogenous antioxidant systems.
(iii) A clear protection of DNA integrity (potential to reduce DNA damage or to increase the resistance of DNA to routine challenges) was detected in the second month for all the inclusion levels, though the most prominent benefit was achieved for the highest level (10%). Again, complex genoprotective pathways were disclosed, involving an intermediary step (expressed in the first month) where a genotoxic trigger (regarded as a pro-genoprotective mechanism) was identified.
(iv) The use of *Salicornia*-enriched formulations as complementary feeds with functional properties showed potential, but the choice of dose and supplementation time tended to be critical.

In short, these results highlight a promising solution to improve aquaculture practices, also unraveling a complementary novel, low-value raw material, and a valorization pathway.

## Figures and Tables

**Table 1:** **Main ingredients of the experimental diets:** standard feed or control (C) and feeds with *S. ramosissima* incorporation at 2.5% (S2.5), 5% (S5), and 10% (S10).

Ingredients (%)	Diets			
	C	S2.5	S5	S10
Fish meal LT70 ^1^	35.0	35.0	35.0	35.0
Kill meal ^2^	5.0	5.0	5.0	5.0
Soy protein concentrate ^3^	13.0	13.0	13.0	13.0
Wheat gluten ^4^	10.0	10.1	10.1	10.3
Corn gluten meal ^5^	8.0	8.0	8.0	8.0
Wheat meal ^6^	16.3	13.7	11.2	6.0
Vitamin and mineral premix ^7^	1.0	1.0	1.0	1.0
Monocalcium phosphate ^8^	0.8	0.8	0.8	0.8
Fish oil ^9^	5.2	5.2	5.2	5.2
Rapeseed oil ^10^	5.7	5.7	5.7	5.7
Salicomia	-	2.5	5.0	10.0

^1^ LT70 steam dried. 70.7% crude protein (CP). 8.1% crude fat (CF). Pesquera Diamante. Peru. ^2^ Krill meal: 52% CP, 22% CF, Aker Biomarine, Norway. ^3^ Soycomil P: 63% CP. 0.8% CF. ADM. The Netherlands. ^4^ VITAL: 83.7% CP. 1.6% CF. ROQUETTE Frères. France. ^5^ Corn gluten meal: 61% CP. 6% CF. COPAM. Portugal. ^6^ Wheat meal: 10.2% CP; 1.2% CF. Casa Lanchinha. Portugal. ^7^ 20 PREMIX Lda. Portugal. Vitamins (IU or mg/kg diet): DL-alpha tocopheryl acetate 100 mg; sodium menadione bisulphate 25 mg; retinyl acetate 20.000 IU; DL-cholecalciferol 2.000 IU; thiamine 30 mg; riboflavin 30 mg; pyridoxine 20 mg; cyanocobalamin 0.1 mg; nicotinic acid 200 mg; folic acid 15 mg; ascorbic acid 500 mg; inositol 500 mg; biotin 3 mg; calcium panthotenate 100 mg; choline chloride 1.000 mg; betaine 500 mg. Minerals (g or mg/kg diet): copper sulfate 9 mg; ferric sulfate 6 mg; potassium iodide 0.5 mg; manganese oxide 9.6 mg; sodium selenite 0.01 mg; zinc sulfate 7.5 mg; sodium chloride 400 mg; excipient wheat middlings.^8^ 21.8% phosphorus, 18.4% calcium, Fosfitalia, Italy. ^9^ 98.1% CF (16% EPA; 12% DHA), Sopropêche, France. ^10^ Henry Lamotte Oils GmbH. Germany.

**Table 2:** **Analyzed proximate composition of the experimental diets:** standard feed or control (C) and feeds with *S. ramosissima* incorporation at 2.5% (S2.5), 5% (S5), and 10% (S10).

% of Dry Matter	Diets			
	C	S2.5	S5	S10
Dry matter	94.1	96.5	95.5	94.5
Crude protein	52.4	51.4	53.7	52.1
Crude lipids	16.8	16.8	17.3	17.3
Ash	9.0	9.8	10.5	11.3
Energy (KJ g^−1^ DM)	23.4	23.1	23.3	22.7

## Data Availability

Data are contained within the article.
